# Deep and Clear Optical Imaging of Thick Inhomogeneous Samples

**DOI:** 10.1371/journal.pone.0035795

**Published:** 2012-04-25

**Authors:** Raphael Jorand, Gwénaële Le Corre, Jordi Andilla, Amina Maandhui, Céline Frongia, Valérie Lobjois, Bernard Ducommun, Corinne Lorenzo

**Affiliations:** 1 University of Toulouse, ITAV-UMS3039, Toulouse, France; 2 CNRS, ITAV-UMS3039, Toulouse, France; 3 ICFO, Institut de Ciences Fotonique, Mediterraneen Technology Park, Castelldefels, Barcelona, Spain; 4 CHU de Toulouse, Toulouse, France; Johns Hopkins University, United States of America

## Abstract

Inhomogeneity in thick biological specimens results in poor imaging by light microscopy, which deteriorates as the focal plane moves deeper into the specimen. Here, we have combined selective plane illumination microscopy (SPIM) with wavefront sensor adaptive optics (wao). Our waoSPIM is based on a direct wavefront measure using a Hartmann-Shack wavefront sensor and fluorescent beads as point source emitters. We demonstrate the use of this waoSPIM method to correct distortions in three-dimensional biological imaging and to improve the quality of images from deep within thick inhomogeneous samples.

## Introduction

Understanding the hierarchical organization of multi-protein complexes, organelles and networks at a cellular level within integrated biological systems is one of the major challenges of modern biology. There is a genuine need for innovative tools that can rapidly provide high spatial and temporal resolution 3D images of thick biological specimens [1]. Selective plane illumination microscopy (SPIM) is an emerging technology proposed to solve this problem. Its direct optical sectioning can be used in a large variety of live biological samples to allow visualization of fluorescent signals with low photo- toxicity, high temporal resolution and good penetration depth imaging [2]–[5]. It uses a sheet of light to illuminate the sample at an angle of 90 degrees to the detection axis. The light sheet is positioned in the focal plane of a detection microscope objective. The resolution in the plane is equivalent to that of a widefield microscope; the finite extent of the light sheet in the z-axis allows effective optical sectioning. SPIM has been successfully applied on «semi transparent» model organisms, such as Zebrafish, Medaka and Drosophila and has been shown to achieve approximately 6 µm axial resolution in thick samples up to a depth of about 500 µm over a field of view ranging between 0.04–2 mm^2^ [2]. Furthermore few studies reported the use of SPIM to image thick, inhomogeneous and highly scattering specimens such as multicellular tumor spheroid (MCTS) [6]–[7]. Although SPIM is well adapted to imaging those samples at subcellular resolution, it suffers from the optical aberrations induced by the specimen as any other light microscopy technique. Spatial variations in the refractive index of the specimen (due to cell membranes, fat deposits and extracellular matrix components, for example) cause major changes to the light path, resulting in aberrant images [8]–[10]. These effects are particularly obvious when thick inhomogeneous, biological specimens are investigated; loss of signal and contrast in the deepest regions of the sample thus impairs the in-depth imaging capability.

Adaptive optics (AO), a method originally developed for astronomical telescopes, measures and corrects aberrations, thus restoring the optimum performance of the imaging system [11]. It does so by introducing a sensor, such as a Hartmann–Shack wavefront (HSWF) sensor, which measures the distorted wavefront coming from an ideal point source emitter. In astronomy, the light coming from a well-known star (guide star) or from the fluorescence produced when focusing a powerful pulsed-laser in the high atmosphere (artificial guide star) is often used. From the measure of the wavefront, a corrective element, such as a deformable mirror (DM), compensates the optical aberrations by means of a closed feedback loop.

A point source emitter, similar to the “guide star" used in astronomy, is a prerequisite for analyzing a wavefront with an HSWF sensor. Because biological samples usually do not have a natural point-source emitter, and due to the difficulty of inserting a wavefront sensor in the optical path of a microscope, most AO microscopes were sensorless systems [12], [13]. These systems, based on iterative algorithms, were intrinsically slow and usually required numerous iterations that led to photo- bleaching and photo-toxicity. Recently, Azucena and coworkers [14] introduced fluorescent microspheres into thin samples (up to 45 µm) as fluorescence point source emitters thus allowing the measurement of the wavefront and its correction with an AO confocal microscope [15].

In this report, we describe a straightforward approach in SPIM for the use of wavefront sensor adaptive optics (wao) to correct imaging path and improve the quality of SPIM imaging in depth using fluorescent beads as ideal point source emitters. The implementation of an AO loop in confocal microscopy is complex because of the collinear geometry of the illumination and detection pathways. In contrast SPIM is well adapted to implement such a device because the optical paths of illumination and detection operate independently. We take advantage of this inherent characteristic of the SPIM architecture to correct the detection path without affecting the illumination path, which needs a different treatment because of its perpendicular incidence. Furthermore, the light sheet and detection objectives are fixed, facilitating the conjugation of the DM and the HSWF microlens array to the detection lens pupil. In order to validate the waoSPIM, we performed experiments using fluorescent beads on different phantom preparations, which demonstrates that our waoSPIM could accurately correct substantial aberrations. Finally, we also demonstrate the image improvement and imaging depth enhancement in fixed, large (of about 400 µm diameter) MCTS stably expressing fluorescent biomarkers.

## Results

### Experimental setup

**Figure 1:**
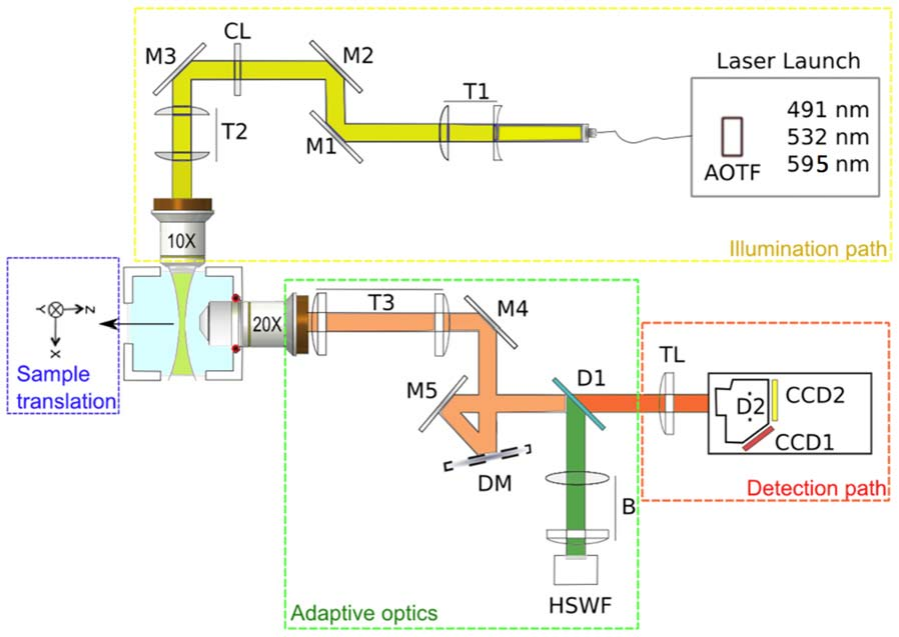
Scheme of the waoSPIM setup. In our experimental setup (Fig. S1), a cylindrical lens focuses the light to a horizontal line (light sheet) that is imaged into the back focal plane of an illumination objective (10× NA 0.25) (yellow path). The sample is positioned in the light sheet inside a physiological chamber filled with aqueous medium. The emitted light is collected (red path) by an immersion objective (20× NA 0.5) fitted to the physiological chamber. AOTF, acousto-optic tunable filter; T1–T3, telescopes; M1–M5, mirrors; CL, cylindrical lens; DM, deformable mirror; D1 and D2, dichroic mirrors; HSWF, Hartmann–Shack wavefront sensor; B, Lens system for DM-HSWF pupil conjugation; TL, tube lens; CCD 1 and CCD2, coupled charged devices.

A schematic illustration and photography of the waoSPIM are shown in Fig. 1 and Fig.S1. To avoid the pollution on the sample's image by the fluorescence of beads used as artificial stars, we created a spectral independence between the image of the sample and the image of the fluorescent beads. As a light source, we used a compact laser launch (Errol) comprising the outputs of three diode-pumped solid-state lasers (491 nm, 532 nm and 595 nm) combined by dichroic mirrors into a single multi-wavelength beam. A four-channel acousto-optic tunable filter (AOTF) with a separate blanking channel provided precise control over each laser's illumination intensity. The output of the AOTF was coupled into a fiber. The output of the fiber was collimated and expanded by a fixed telescope (T1). A cylindrical lens (CL) focused the light in one axis that was conjugated to the back pupil plane of the illumination lens (10× NA 0.25, air; Leica Microsystem) by a telescope (T2). A water immersion objective (20× NA 0.5; Leica Microsystem) collected the light emitted from the fluorescent beads and the sample. A telescope (T3) served to conjugate the back pupil plane of this objective onto a deformable mirror (DM) with 52 actuators, a 15 mm effective diameter and a stroke of 50 µm Peak to Valley in tilt (mirao™ 52-e; Imagine Optic). To measure the wavefront directly from the sample, we positioned an HSWF sensor in the detection path of our SPIM setup. The DM was conjugated to the HSWF sensor, composed of a matrix of 32×32 microlenses of 3.6 mm effective diameter (HASO™ 32; Imagine Optic). As point source emitters, we used 2.5 µm fluorescents beads, which were smaller than the limit diffraction of the HSWF sensor and that provided sufficient light to reconstruct the wavefront. The single-edge dichroic beamsplitter (D1, FF 520.Dio1.25×36, Semrock) directed the 488–512 nm signal emissions to the HSWF sensor and the 528–655 nm signal to a simultaneous dual wavelength imaging system that feature two CCD devices (ORCA-D2; Hamamastu). In addition this dual camera offer the advantage of correcting automatically the axial focus shift between both wavelengths. A “Stop Line" filter (not shown) had been positioned in the detection path before the HSWF sensor in order to reject the 491 nm laser line. We also take advantage of the fact that the fluorescent beads had a large fluorescence emission spectrum and can be simultaneously imaged by the HSWF sensor and the CCD2 camera. The CCD1 camera imaged the sample, which was stained with another fluorophore. The sample holder can be moved in any dimension (x, y, z) by fully motorized stages.

### AO loop

**Figure 2:**
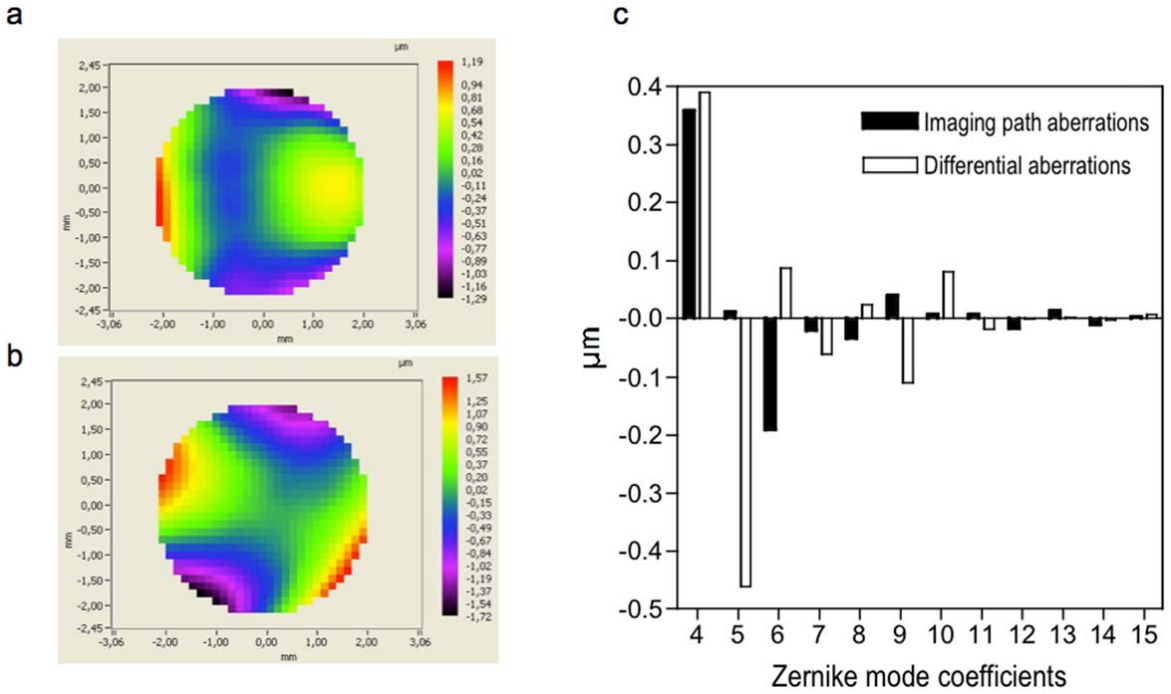
Non-common path optical aberrations. (a) Recorded wavefront in the imaging camera focal plane corresponding to the imaging path aberrations. (b) Reference recorded wavefront in the HSWF sensor path after correction of the imaging path aberrations corresponding to the differential aberrations. (c) Graph showing the 3^rd^ and 5^th^ order of Zernike coefficients of (a) and (b).

The procedure for the AO loop correction started by positioning a fluorescent bead in the center of the field of view of the detection objective. To correctly measure the wavefront, a diaphragm allowed collecting the fluorescence emitted only by the selected bead. When the AO loop was turned on, a software closed-loop algorithm minimized the wavefront errors by analyzing the signal from the HSWF sensor and by calculating the appropriate DM shape to compensate for the measured wavefronts. In the closed-loop algorithm implemented in the software CASAO™, the information from the HSWF sensor is decomposed into the orthogonal modes of the DM by using a standard algorithm of singular value decomposition. A simple linear equations problem is solved to find the best shape of the mirror to compensate for the aberrations. In the case of the DM mirao™ 52-e, because of its linearity, the equations system is reduced to a simple subtraction. In our experimental conditions, the system achieved an effective closed-loop bandwidth of 5 Hz and one or two iterations were needed to converge to a correct wavefront. In waoSPIM system, the dichroic beam splitter D1 (Fig. 1) is one of the most critical elements because it introduced aberrations, especially astigmatism and coma aberrations. Prior to experiments, we measured these “static" aberrations due to the non-common path between the HSWF sensor and the dual CCD by positioning the HSWF sensor in the imaging camera focal plane (Fig. 2a) and then compensated for these aberrations using the AO loop system. The purpose of this step was to set the DM shape such that it corrects the imaging path aberrations. We will refer to this DM shape as “without AO" in the following sections. Then, by re- positioning the HSWF sensor on its path, a reference wavefront (Fig. 2b) was then recorded that corresponds to the differential aberrations. Figure 2c shows the 3^rd^ and 5^th^ order of Zernike coefficients for the wavefronts shown in Fig. 2a and Fig. 2b. As we mentioned before, the differential aberrations between the HSWF sensor and the imaging path are essentially astigmatism, coma and trefoil. These aberrations are mainly due the dichroic beam splitter D1. In the AO closed loop, the reference wavefront (Fig. 2b) was used to target all the aberrations and the initial measured wavefront from the bead was used to target the tip/tilt.

### waoSPIM performance

**Figure 3:**
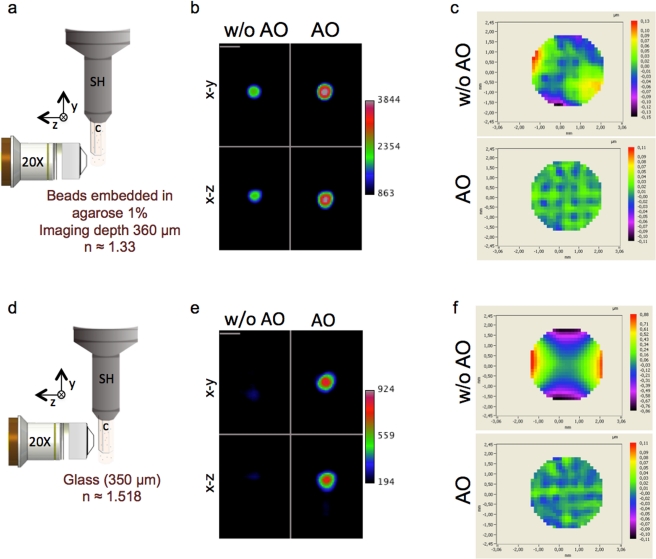
waoSPIM performance. a, b and c correspond respectively to the sketch of the phantom beads geometry, the profile views and the wavefront maps for beads embedded in agarose 1% and imaged at a depth of 360 µm. d, e and f correspond respectively to the sketch of the phantom beads geometry, the profile views and the wavefront maps for beads embedded in 1% of agarose and imaged through the capillary glass at a depth of 350 µm. Images were acquired either without (w/o AO) and with AO (AO) at a fixed excitation intensity at 491 nm and a 100 ms exposure time. (a, d) In Sketches SH, sample holder; C, capillary. (b, e) Profile views without (w/o AO) and with AO (AO) of the maximum intensity projections x-y and x-z for beads either embedded in a cylinder of agarose (b), or in agarose imaged through capillary glass (e). Scale bar, 5 µm. (c, f) Wavefront maps corresponding to the raw wavefront minus reference wavefront recorded with the DM shape set to correct the optical setup aberrations (w/o AO) or with the AO closed loop (AO), the color scale correspond to the wavefront error in µm.

To evaluate the ability of the AO closed loop to correct substantial aberrations in SPIM, we measured the Strehl ratio (an indicator of the AO loop system performance), by applying the Maréchal approximation in several experimental conditions that introduced aberrations into the system (Fig. 3). First, fluorescent beads were embedded in a cylinder of 1% agarose and imaged at depth (360 µm) without or with AO (Fig. 3 ab). In these conditions, the main aberration was essentially astigmatism. When the AO was turned on, we observed a 40% increase in signal intensity. The root- mean- square (RMS) wavefront error was 0.042 µm before correction and was reduced to 0.011 µm after correction (Fig. 3c). The values for full width at half-maximum (FWHM) were near the performance limit of SPIM (Table 1). The Strelh ratio improved from *S* = 0.756±0.04 (sem) to *S* = 0.974±0.004 (sem). In the second experiment, beads were embedded in agarose and imaged through the glass of a capillary (Fig. 3 de), which introduced a variety of aberrations, including coma, astigmatism and spherical aberrations (Fig. 3f). The image of the beads was completely deformed laterally and axially. AO restored the correct image of the beads (Fig. 3e). The signal intensity increased by 70% and near-diffraction-limited performance was again achieved (Table 1). The RMS wavefront error reduced from 0.374 to 0.011 µm (Fig. 3f). After correction, the Strehl ratio was *S* = 0.982±0.001(sem).

**Table 1:** waoSPIM performance.

	Agarose		Glass	
	*w/o* AO	AO	*w/o* AO	AO
FWHM x (µm)	3.164	2.851	8.754	3.24
FWHM y (µm)	3.041	2.875	9.514	3.102
FWHM z (µm)	2.885	2.969	3.0	3.128
RMS (µm)	0.042	0.011	0.374	0.011
Strelh ratio	0.769	0.983	na	0.982
Strelh ratio mean (n>10)	0.756	0.974	na	0.982

Values obtained for the full width at half maximum (FWHM), the root-mean-square (RMS) and Strelh ratio from the bead images and wavefront map in Figure 2. The FWHM was determined from the parameters retrieved from the fitted curve obtained from the plot intensity profile along the three axes respective to views in Figure 2 b and e. na, not applicable.

In both experiments, AO correction increased the signal intensity and improved the lateral FWHM. By contrast, the values for the axial FWHM were not greater after correction than before. This effect could be explained by the fact that AO, in this optical configuration, do not correct the light sheet illumination that defines the axial resolution of the system.

These data show that waoSPIM is effective and can correct substantial aberrations accurately.

### Signal and contrast improvement when imaging in depth with AO correction

**Figure 4:**
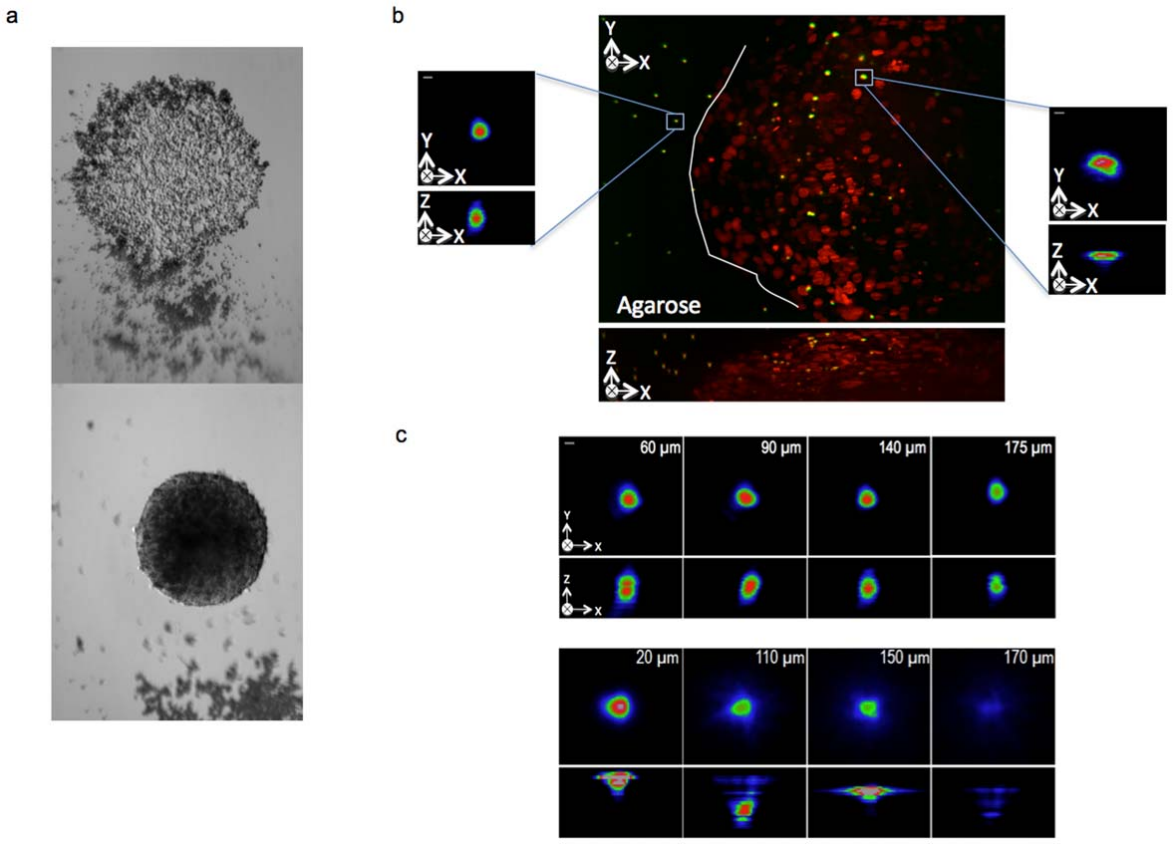
MCTS induced aberrations. (a) Transmitted light images of cells seed with 2.5 µm green beads (InSpeck Green, I-7219, Invitrogen) in a 96- well plate (bottom image). After 4 days in culture, these cells formed a MCTS incorporating the beads (below image). (b) Maximum projection of a three-dimensional stack of 100 images (z spacing 1 µm) of a MCTS expressing a fluorescent nuclear protein, H2B–HcRed and cultivated in presence of green fluorescence beads as shown in (a). Insets show magnified views of the bead outside (left) or inside (right) the spheroid. Scale bar, 2 µm. (c) Profile views of the maximum intensity projections x-y and x-z for beads either outside (top panel), or inside the MCTS (bellow panel) imaged at different depths. Images were acquired at fixed excitation intensity at 491 nm and a 100 ms exposure time. Images share the same intensity scale. Scale bar, 2 µm.

MCTSs are 3D culture models [16] that present attractive advantages to investigate the influence of malignant cell interactions during cell proliferation; however, they raise significant challenges for imaging by light microscopy. With such thick, non-transparent biological samples, specimen-induced aberrations are significant (Fig. 4). To define the form and the magnitude of aberrations introduced by the MCTS, we imaged fluorescent beads integrated during the culture inside the MCTS (Fig. 4a, b) and recorded their wavefront maps. We found that astigmatism, coma and trefoil terms were the dominant aberrations induced by the MCTS (Fig. S2) and their amplitudes increased with imaging depth into the MCTS (Fig. 4c). To evaluate our waoSPIM setup to improve image quality by correcting the aberrations introduced by the MCTS, we imaged fixed large (400 µm diameter) MCTS stably expressing a fluorescent nuclear protein, Histone H2B–HcRed. As shown in Fig S3 the waoSPIM in “AO off" position give similar results than a conventional SPIM [7] in term of image quality and penetration depth. We defined a suitable image contrast measure based on the image gradient (*IG*) to compare the effects of the AO correction on image quality. The image has been divided in square Regions Of Interest (ROIs) and the IG has been determined in each ROI. In order to correctly sample the studied objects, each ROI should not be more than half of the size of the object. Because of this, the lateral size of ROI's (*n*) has been set to 36 pixels, which corresponds to the half of the typical diameter of a nucleus in the image. For each ROI, we determined the IG as follows:
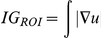
where *u* is the intensity of the ROI. This integral in the discrete form becomes
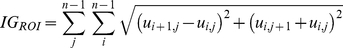
where (i, j) are the horizontal and vertical indices of each pixel in each ROI.

This definition of IG provides a quantification of the variations inside the ROI, which represents a quantification of the image contrast. Thus, by using this method, the improvement achieved can be objectively measured as the ratio between each corresponding ROI.

**Figure 5:**
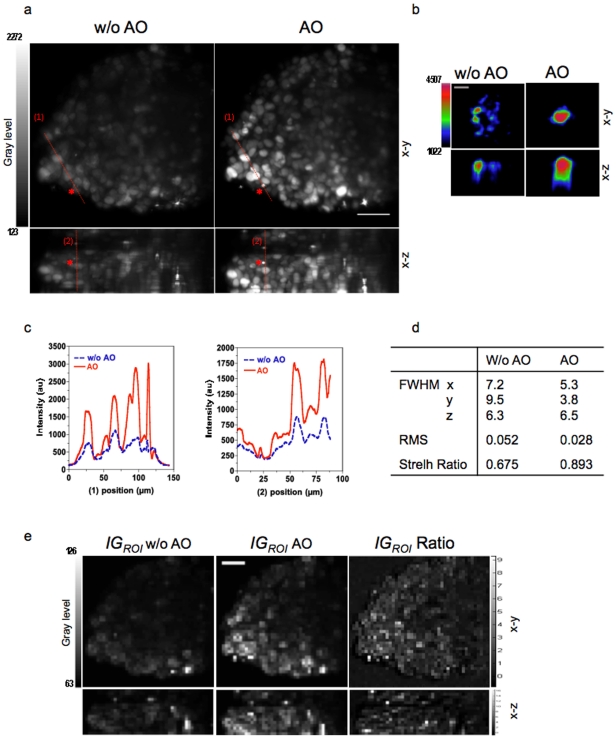
Signal and contrast improvement. Maximum projection of a 3D stack of 100 images (z spacing 1 µm) of a large MCTS expressing a fluorescent nuclear protein, H2B–HcRed, without (w/o AO) and with AO (AO). Scale bar, 50 µm. The asterisk marks the bead used as the point source emitter located at a depth of 150 µm. Both images were acquired by using the same excitation intensity at 595 nm and a 300 ms exposure time. (b) Magnified views of the bead. Scale bar, 5 µm. (c) Intensity profiles along the lines 1 and 2 indicated in (a). (d) FWHM (µm), RMS and Strelh ratio values for bead images in (b). (e) *IG_ROI_* mapping images calculated from images in (a) and *IG_ROI_* Ratio image calculated as
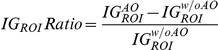
where 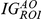 is 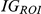 mapping images calculated from images obtained with AO and 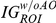 is 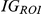 mapping images calculated from images obtained without AO.

**Figure 6:**
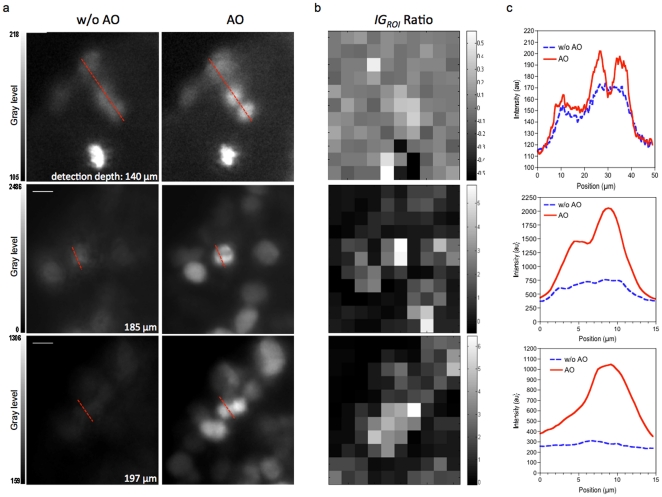
Image quality improvement in depth. (a) Single planes images of nuclei and mitotic figures at various depths within the MCTS resolved with AO or not (w/o AO). Images are acquired by using the same excitation intensity at 595 nm and same exposure time (300 ms). Scale Bar 5 µm. (b) The corresponding *IG_ROI_* Ratio image. (c) The corresponding intensity plots along the line indicated.

The images obtained with AO were much brighter and had higher contrast than images acquired without AO (Fig. 5 and Movies S1 and S2). For the bead, AO increased the intensity by 40% and reduced the RMS wavefront error to 0.028 µm (Fig. 5b,d). Nuclei that were not well resolved without AO were resolved when the method was applied (Fig. 6a). The gain in information is clearly illustrated by images of mitotic cells deep in the MCTS, which were resolved well with AO whereas they were not easily identified without (Fig. 6 b–c). Furthermore AO correction is also effective in the deeper layers of the MCTS, up to 200 µm in depth (Fig. 6). It is worth to notice that this image quality improvement could not be obtained by increasing the excitation power or acquisition exposure time. The AO correction provided significant improvement in the intensity signal and in the contrast of the images. This improvement was sufficient to perform nuclei segmentation and 3D reconstruction from deep inside the MCTS (Fig. S4 and Movies S3 and S4).

## Discussion

Variations in refractive index due to the different components of a tissue limit the performance of microscopy on whole tissues and organisms. These effects are more pronounced in thick tissues. SPIM is a useful technique for imaging large and dense biological samples at high spatial resolution. Although very promising, SPIM technology still requires improvements before it can be used to image cells deep in thick samples. We have applied wao to SPIM and demonstrated the ability of this combined waoSPIM technology to correct complex aberrations.

The wavefront measurements from biological samples are usually difficult to obtain since there are no natural point source reference such as the «guide star» used in astronomy. We addressed this issue by measuring directly the wavefront using fluorescent beads directly inserted within the sample as suitable fluorescence point source references. The fluorescent beads were easily integrated during the MCTS culture and we have demonstrated that this method allowed a direct measurement of aberrations induced by MCTS and their compensation by an adaptive optics closed loop. Our data demonstrate improved image signal intensity and contrast, sufficient to identify mitotic cells deep inside the spheroids. Even if fluorescent beads did not seem to alter the MCTS growth, they could disturb the cell behaviors inside the MCTS and so the natural MCTS architecture. These possible effects should be considered for future living 3D imaging MCTS studies. Furthermore, beads are randomly distributed inside the MCTS. However each correction would be effective for a given region around each bead in the three dimensions axes (x, y and z). The extent of this region would depend on the heterogeneity of the surrounding volume. Then, parameters such as heterogeneity of the sample, the bead's density and/or distribution could limit the quality of the overall correction. To overcome these difficulties and to provide a minimally invasive and more versatile method, we will aim at developing genetically encoded fusion fluorescent biomarkers distributed in the whole MCTS that will be used as biological «guide stars».

A major benefit of SPIM is the fast data acquisition, the speed of image acquisition in waoSPIM is limited by the exposure time and sampling required to measure accurately the wavefront from the «guide star». In our experimental conditions and for beads inside a MCTS, the wavefront sensor requires 100–300 ms for each measurement and this exposure time varies depending on the depth at which one images. With a closed loop bandwidth of approximately 5 Hz and one or two iterations needed to converge to a corrected wavefront, the image acquisition speed of the MCTS sample is only slightly reduced with AO. For applications requiring high-speed acquisition, waoSPIM could be improved by designing a HSWF sensor coupled with a sensitive and fast camera such as an Electron Multiplying CCD (EMCCD).

SPIM is well suited for multi view data acquisition. This is performed by acquiring multiple 3D stacks from different viewing angles, which are then processed by registration and multi view fusion algorithms to combine the different data into a single, high quality three- dimensional image. For inhomogeneous, thick and highly scattering sample, it has been shown that multi view imaging fusion allows retrieving the information from different regions of the sample [17]. Furthermore, the multi view imaging fusion can notably improve the axial resolution and isotropy in images [18]. Therefore, we believe that the application of the multi view imaging fusion modality to waoSPIM is a promising direction for high- quality 3D imaging and high depth imaging of dense and thick samples such as MCTS.

We are convinced that in the future such microscope will allow researchers to image with high temporal and spatial resolution many important biological processes occurring deep inside tissues.

## Materials and Methods

### Cell culture

We constructed a pcDNA3 plasmid (Invitrogen) encoding an H2B–tandem HcRed fusion protein and a hygromycin resistance gene. The HCT116 cell line stably expressing the H2B–HcRed fusion protein (HCT116-H2B-HCRed) was obtained by transfection of HCT116 cells (ATCC number: CCL-247™) with this plasmid by using Jet PEI reagent (Polyplus Transfection) (GMO authorization number 5702). HCT116-H2B–HCRed cells (were cultured in DMEM+GlutaMAX (Dulbecco's Modified Eagle Medium; Gibco) supplemented with 10% fetal bovine serum and 1% penicillin–streptomycin (Pen Strep; Gibco), and maintained at 37°C and 5% CO_2_ in an incubator.

MCTS were prepared in 96-well plates that were coated with 20 mg/ml polyHEMA (Sigma). Cells were plated at a density of 600 cells/well in 100 µl cell culture medium per well then centrifuged to allow spheroid formation. When necessary, 2.5 µm fluorescent beads (Invitrogen Inspeck Green 505/515 100% relative intensity) were added to the cells before centrifugation. After 4–5 days growth, MCTS of 400–500 µm diameter were collected, washed three times with PBS and then fixed with 10% neutral buffered formalin (Sigma–Aldrich) at room temperature for 4 hours. MCTS in formalin were washed three times and stored in PBS.

### Sample preparation

Beads and MCTS samples were embedded in 1% agarose (Euromedex Low Melting Point) in 50 µL capillary pipettes (Hirschmann Laborgeräte Ringcaps).

### Image processing

Images were processed with the open-source image-processing package Fiji. 3D visualization and 3D reconstructions were performed with IMARIS 7.0.0 software (BitPlane inc.).

## Supporting Information

Movie S1 3D volume rendering of H2B-HcRed MCTS stack obtained without AO
(MOV)

Movie S2 3D volume rendering of H2B-HcRed MCTS stack obtained with AO
(MOV)

Movie S3 3D reconstruction of H2B-HcRed MCTS stack obtained without AO
(MOV)

Movie S4 3D reconstruction of H2B-HcRed MCTS stack obtained with AO
(MOV)

Figure S1 waoSPIM setup. Photograph of waoSPIM. The figure shows the illumination path (yellow dashed line), the wavefront analysis path (green dashed line) and detection path (red dashed line).
(TIF)

Figure S2 MCTS induced aberrations. Graph shows the 3^rd^ and 5^th^ order of Zernike coefficients from beads inside the MCTS. The error bars represent the standard error of mean. (n>15)
(TIF)

Figure S3 Comparison between conventional SPIM and waoSPIM in “AO off." Maximum projection of a three-dimensional stack of 100 images (z spacing 1 µm) of a multicellular tumor spheroid expressing a fluorescent nuclear protein, H2B–HcRed, imaged by a conventional SPIM (a) or by waoSPIM in “AO off" (b). Scale bar, 50 µm. Insets show magnified views of mitotic cell. Scale bar, 5 µm.
(TIFF)

Figure S4 3D reconstruction improvement. Three-dimensional reconstruction of the stack of images shown in Figure 5 of an MCTS expressing H2B-HcRed and cultivated in presence of green fluorescent beads, w/o AO (a) and with AO (b). Scale bar, 40 µm. (c–f) Magnification of the region outlined in a and b. Scale bar 15 µm (cd), 10 µm (ef)). Red isosurfaces correspond to interphase nuclei, green isosurface to the “guide star" bead and the yellow surface to mitotic condensed chromosome. Three- dimensional reconstructions were performed with Imaris 7.0.0 software. Surfaces were reconstructed with the smooth option, a surface area detail level of 0.680 and “enable eliminate background = true", excepted for bead (value = false). Nuclei surfaces were reconstructed with a diameter of largest sphere value of 2.55 µm and a threshold ratio of 0.05 (87.399 µm^2^ with a maximum of 1718 µm^2^) for AO stack and a ratio of 0.07 (37.988 µm^2^ with a maximum of 528 µm^2^) for w/o AO stack. A filter was used on both stacks to remove particles with a volume less than 90 µm^3^. The surface of the bead (in green) was reconstructed with a ratio of 0.32 (744.867 µm^2^ with a maximum of 2338 µm^2^) for AO stack. Due to noise and variation of intensity, the surface of the bead for w/o stack was reconstructed in two parts with ratio of 0.34 (411.981 µm^2^ with a maximum of 1196 µm^2^) and 0.29 (341.482 µm^2^ with same maximum). The surface of mitotic chromosome mass (in yellow) was reconstructed with a diameter of largest sphere value of 0.3 µm and a ratio of 0.04 (3.277 µm^2^ with a maximum of 82 µm^2^) for AO stack and with a diameter of largest sphere value of 1 µm and a ratio of 0.07 (18.616 µm^2^ with a maximum of 255 µm^2^) for w/o AO stack.
(TIF)

## References

[1] Ntziachristos V (2010). Going deeper than microscopy: the optical imaging frontier in biology.. Nat Methods.

[2] Huisken J, Swoger J, Del Bene F, Wittbrodt J, Stelzer EHK (2004). Optical sectioning deep inside live embryos by selective plane illumination microscopy.. Science.

[3] Keller PJ, Schmidt AD, Santella A, Khairy K, Bao Z (2010). Fast, high-contrast imaging of animal development with scanned light sheet-based structured-illumination microscopy.. Nat Methods.

[4] Arrenberg AB, Stainier DY, Baier H, Huisken J (2010). Optogenetic control of cardiac function.. Science.

[5] Sena G, Frentz Z, Birnbaum KD, Leibler S (2011). Quantitation of cellular dynamics in growing Arabidopsis roots with light sheet microscopy.. PLoS One.

[6] Verveer PJ, Swoger J, Pampaloni F, Greger K, Marcello M (2007). High-resolution three-dimensional imaging of large specimens with light sheet-based microscopy.. Nat Methods.

[7] Lorenzo C, Frongia C, Jorand R, Fehrenbach J, Weiss P (2011). Live cell division dynamics monitoring in 3D large spheroid tumor models using light sheet microscopy.. Cell Div.

[8] Schwertner M, Booth MJ, Wilson T (2004). Characterizing specimen induced aberrations for high NA adaptive optical microscopy.. Opt Express.

[9] Kam Z, Hanser B, Gustafsson MGL, Agard DA, Sedat JW (2001). Computational adaptive optics for live three- dimensional biological imaging.. Proc Natl Acad Sci USA.

[10] Simmonds RD, Wilson T, Booth MJ (2011). Effects of aberrations and specimen structure in conventional, confocal and two-photon fluorescence microscopy.. Journal of Microscpy.

[11] Tyson R K (1991). Principles of Adaptives Optics.. Academic.

[12] Debarre D, Booth MJ, Wilson T (2007). Image based adaptive optics through optimisation of low spatial frequencies.. Op Express.

[13] Ji N, Milkie DE, Betzig E (2010). Adaptive optics via pupil segmentation for high-resolution imaging in biological tissues.. Nat Meth.

[14] Azucena O, Crest J, Cao J, Sullivan W, Kner P (2010). Wavefront aberration measurements and corrections through thick tissue using fluorescent microsphere reference beacons.. Opt Express.

[15] Tao X, Fernandez B, Azucena O, Fu M, Garcia D (2011). Adaptive optics confocal microscopy using direct wavefront sensing.. Opt Letters.

[16] Sutherland RM (1998). Cell and environment interactions in tumor microregions: the multicell spheroid model.. Science.

[17] Swoger J, Verveer P, Greger K, Huisken J, Stelzer EH (2007). Multi-view image fusion improves resolution in three-dimensional microscopy.. Opt Express.

[18] Preibisch S, Saalfeld S, Schindelin J, Tomancak P (2010). Software for bead-based registration of selective plane illumination microscopy data.. Nat Methods.

