# Supplementary figures and images for: Deep and Clear Optical Imaging of Thick Inhomogeneous Samples
Source: PLoS One. 2012 Apr 25;7(4):e35795. doi: 10.1371/journal.pone.0035795 (PMC3338470; doi:10.1371/journal.pone.0035795)

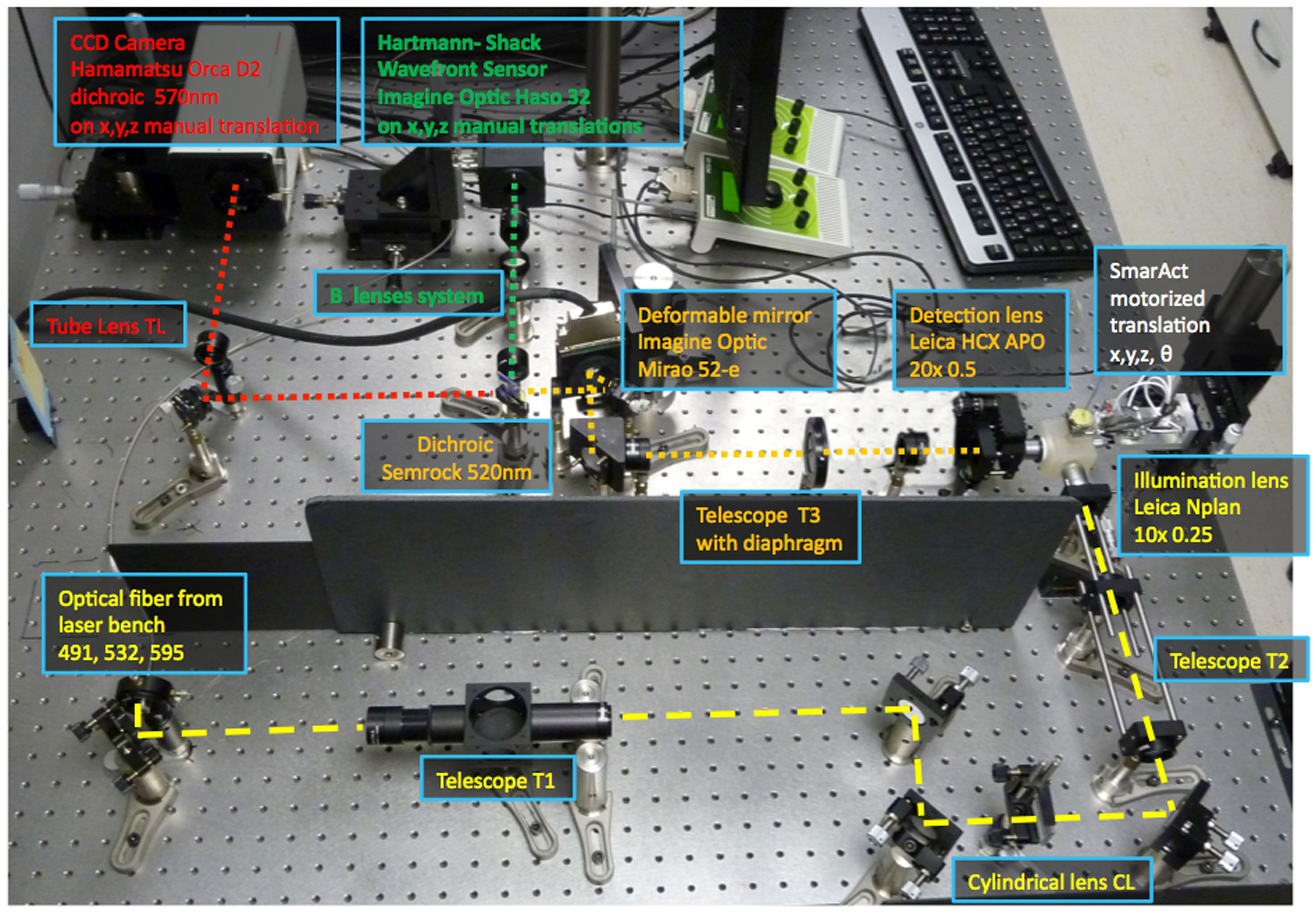

Supplement: Figure S1 — waoSPIM setup. Photograph of waoSPIM. The figure shows the illumination path (yellow dashed line), the wavefront analysis path (green dashed line) and detection path (red dashed line). (TIF) [file pone.0035795.s005.tif]

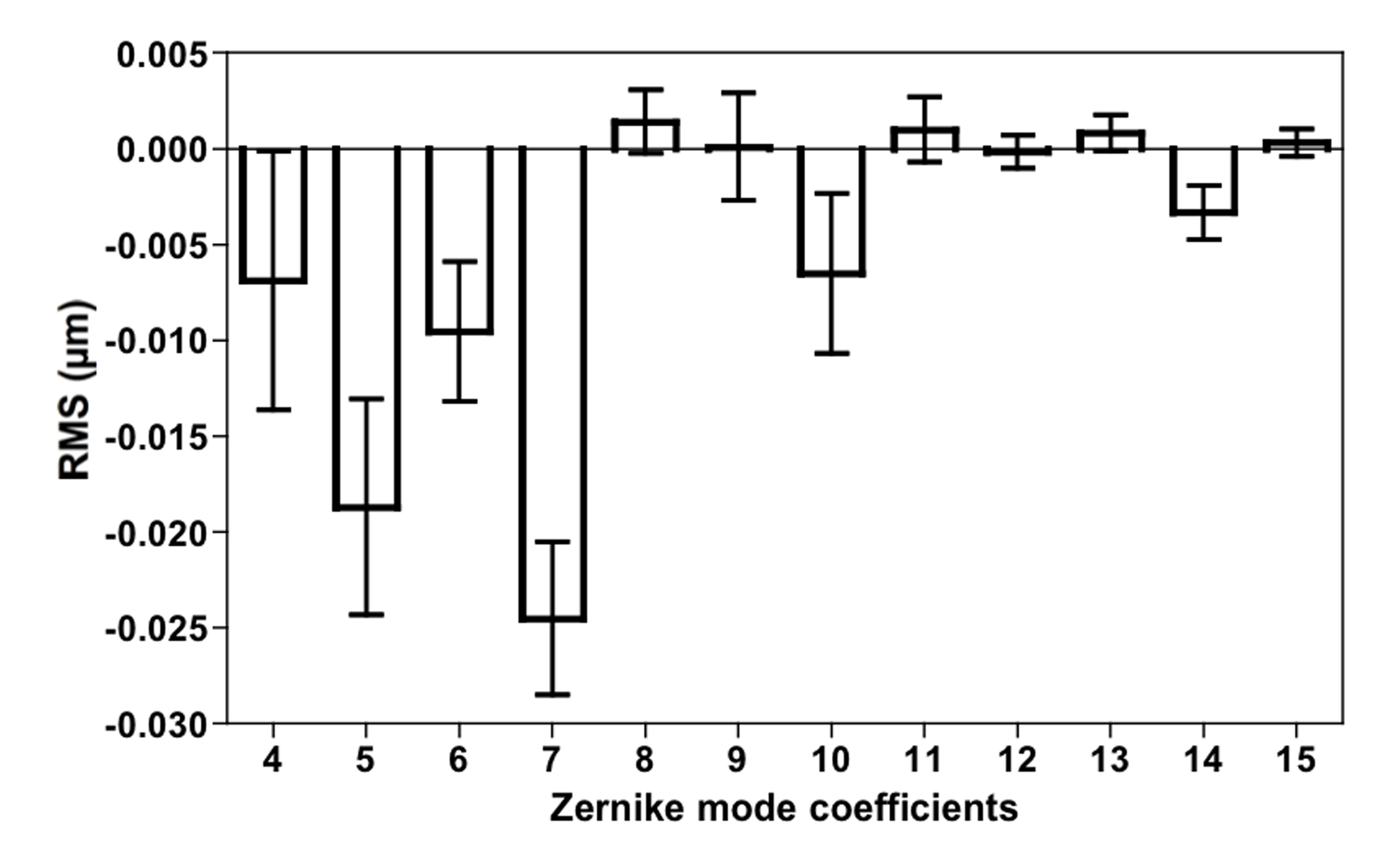

Supplement: Figure S2 — MCTS induced aberrations. Graph shows the 3rd and 5th order of Zernike coefficients from beads inside the MCTS. The error bars represent the standard error of mean. (n>15) (TIF) [file pone.0035795.s006.tif]

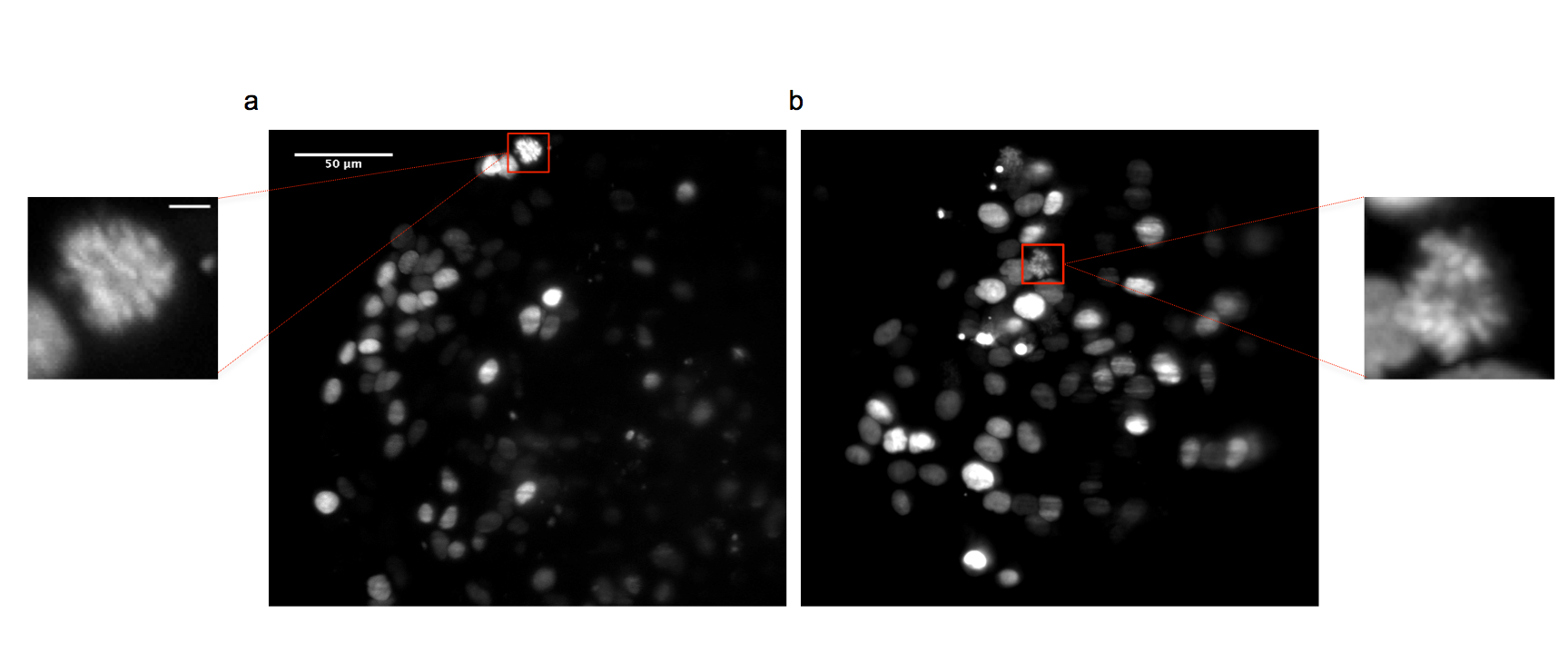

Supplement: Figure S3 — Comparison between conventional SPIM and waoSPIM in “AO off." Maximum projection of a three-dimensional stack of 100 images (z spacing 1 µm) of a multicellular tumor spheroid expressing a fluorescent nuclear protein, H2B–HcRed, imaged by a conventional SPIM (a) or by waoSPIM in “AO off" (b). Scale bar, 50 µm. Insets show magnified views of mitotic cell. Scale bar, 5 µm. (TIFF) [file pone.0035795.s007.tiff]

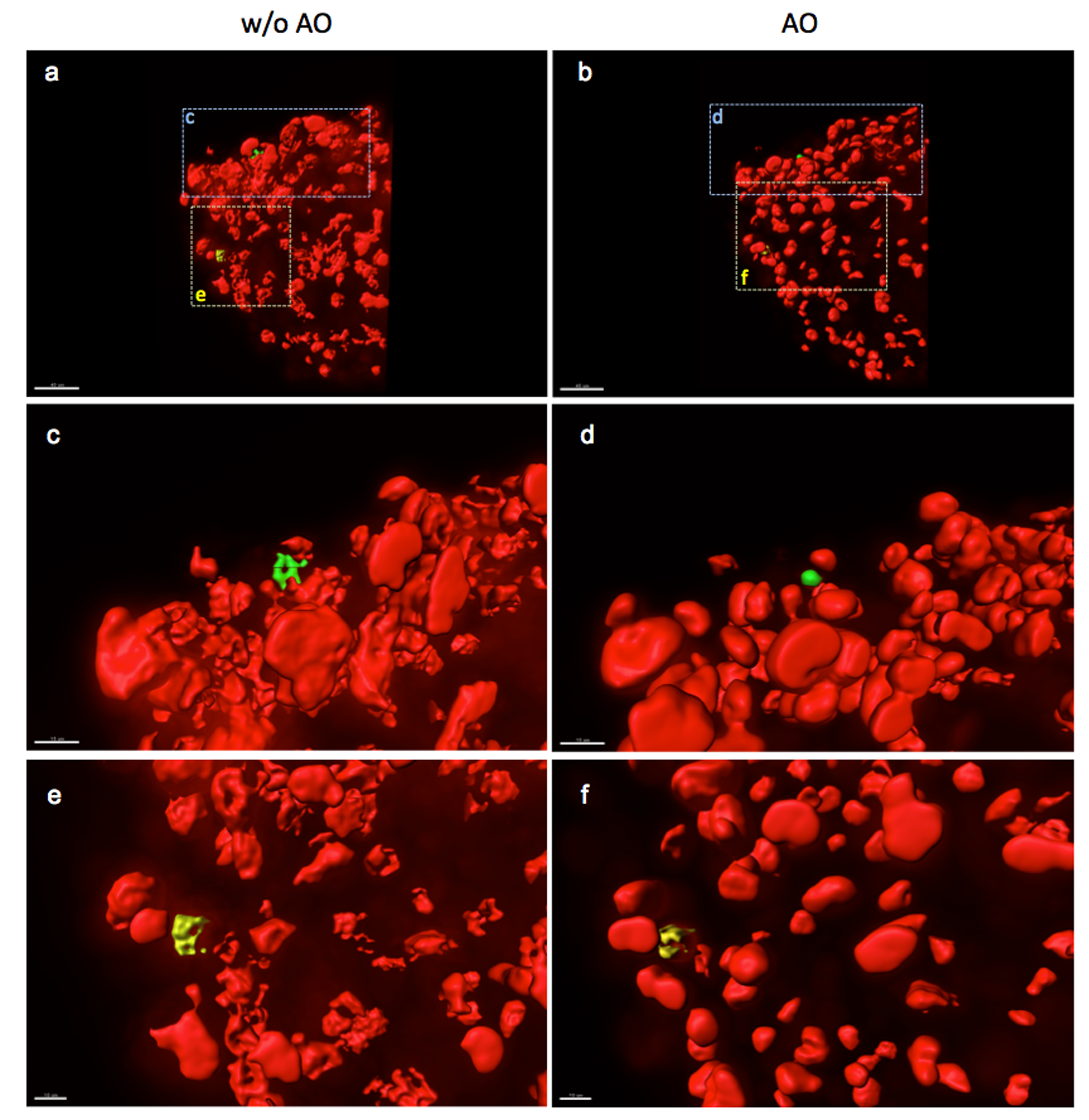

Supplement: Figure S4 — 3D reconstruction improvement. Three-dimensional reconstruction of the stack of images shown in Figure 5 of an MCTS expressing H2B-HcRed and cultivated in presence of green fluorescent beads, w/o AO (a) and with AO (b). Scale bar, 40 µm. (c–f) Magnification of the region outlined in a and b. Scale bar 15 µm (cd), 10 µm (ef)). Red isosurfaces correspond to interphase nuclei, green isosurface to the “guide star" bead and the yellow surface to mitotic condensed chromosome. Three- dimensional reconstructions were performed with Imaris 7.0.0 software. Surfaces were reconstructed with the smooth option, a surface area detail level of 0.680 and “enable eliminate background = true", excepted for bead (value = false). Nuclei surfaces were reconstructed with a diameter of largest sphere value of 2.55 µm and a threshold ratio of 0.05 (87.399 µm2 with a maximum of 1718 µm2) for AO stack and a ratio of 0.07 (37.988 µm2 with a maximum of 528 µm2) for w/o AO stack. A filter was used on both stacks to remove particles with a volume less than 90 µm3. The surface of the bead (in green) was reconstructed with a ratio of 0.32 (744.867 µm2 with a maximum of 2338 µm2) for AO stack. Due to noise and variation of intensity, the surface of the bead for w/o stack was reconstructed in two parts with ratio of 0.34 (411.981 µm2 with a maximum of 1196 µm2) and 0.29 (341.482 µm2 with same maximum). The surface of mitotic chromosome mass (in yellow) was reconstructed with a diameter of largest sphere value of 0.3 µm and a ratio of 0.04 (3.277 µm2 with a maximum of 82 µm2) for AO stack and with a diameter of largest sphere value of 1 µm and a ratio of 0.07 (18.616 µm2 with a maximum of 255 µm2) for w/o AO stack. (TIF) [file pone.0035795.s008.tif]
